# Quantum Zeno-type effect and non-Markovianity in a three-level system

**DOI:** 10.1038/srep39061

**Published:** 2016-12-20

**Authors:** Antti Karlsson, Francesco Francica, Jyrki Piilo, Francesco Plastina

**Affiliations:** 1 Turku Centre for Quantum Physics, Department of Physics and Astronomy, University of Turku, FI-20014 Turun yliopisto, Finland; 2 Dip. Fisica, Universitá della Calabria, 87036 Arcavacata di Rende (CS) Italy; 3 INFN - Gruppo collegato di Cosenza, 87036 Arcavacata di Rende (CS) Italy

## Abstract

We study the coexistence of the quantum Zeno-type effect and non-Markovianity for a system decaying in a structured bosonic environment and subject to a control field. The interaction with the environment induces decay from the excited to the ground level, which, in turn, is coherently coupled to another meta-stable state. The control of the strength of the coherent coupling between the stable levels allows the engineering of both the dissipation and of the memory effects, without modifying neither the system-reservoir interaction, nor environmental properties. We use this framework in two different parameter regimes corresponding to fast (bad cavity limit) and slow dissipation (good cavity limit) in the original and un-controlled qubit system. Our results show a non-monotonic behavior of memory effects when increasing the effectiveness of the Zeno-like freezing. Moreover, we identify a new source of memory effects which allows the persistence of non-Markovianity for long times while the excited state has already been depleted.

How can a flying arrow be moving, if at any instant of time when it is observed, it is seen in some place, stationary? This was one of the paradoxes of Zeno of Elea^1^, an ancient Greek philosopher. A little bit over two thousand years later, von Neumann’s reduction postulate^2^ laid the foundation for a similar effect in quantum mechanics. Namely, as a quantum measurement collapses the state of a quantum system to a state in the measurement basis, if this process is repeated *N* times a second and *N* is let to tend to infinity, the motion of the system is prevented. The quantum phenomenon was named the quantum Zeno effect in ref. 3 and has been studied in several earlier works including also decay in structured reservoirs, see refs 4, 5, 6, 7, 8, 9, 10, 11, 12, 13, 14 and references therein. Whilst Zeno effect was originally associated to frequent measurements, here we use the term in wider context with control fields^12^,^15^,^16^,^17^ which is sometimes called also dynamical control^11^,^18^. Indeed, besides frequent measurements, a strong coupling to an external, control system or level can also prevent the original system of interest from evolving in time^19^,^20^. Loosely speaking the control system is continuously measuring or gazing at the system of interest and thereby preventing its dynamics. This effect is called dominated evolution or the watchdog effect and is the one that we will study in this paper from the point of view of non-Markovianity and information flow. Other studies in the past have discussed modifications of the Quantum Zeno effect in the case of non-Markovian behaviors, see e.g. refs 10,17,21, 22, 23. Here, however, we are interested not only in the Zeno-like physics in presence of memory effects, but also (and more prominently) in the modifications of the non-Markovian character of the dynamics induced by the Zeno-type mechanism; and, to this end, at variance with previous works, we will evaluate non-Markovianity in a quantitative way.

In general terms, Markovian dynamics is typically understood to be describable by the semigroup evolution and Lindblad equation^24^. However, Markovian dynamics is always an approximation and not necessarily valid for all systems nor environments. Therefore, understanding non-Markovian memory effects is an important aspect when studying open system dynamics in general. Different approaches to define and quantify non-Markovian dynamics based on different physical or mathematical quantities have been actively developed in recent years^25^,^26^,^27^,^28^,^29^,^30^, see also recent reviews^31^,^32^. These quantifiers are based on the non-monotonicity of some kind of information flow towards the system, and have been compared and classified in refs 28,33, 34, 35, 36, 37, 38.

In this paper, we will focus on the modifications of the information flow due to the control field induced freezing of the decay, and, more generally, on the possibility to control non-Markovianity by the Zeno effect. For this purpose, we study a two-level system interacting with a zero-temperature bosonic environment. In this qubit system, the coupling strength to the environment and environmental properties, such as spectral density, define whether the qubit dynamics displays memory effects or not. However, adding a coherent coupling to an auxiliary third level allows to control the excited state dynamics – displaying Zeno-like effect– and also to engineer the memory content of the dynamics.

## Model and Dynamics

**Figure 1:**
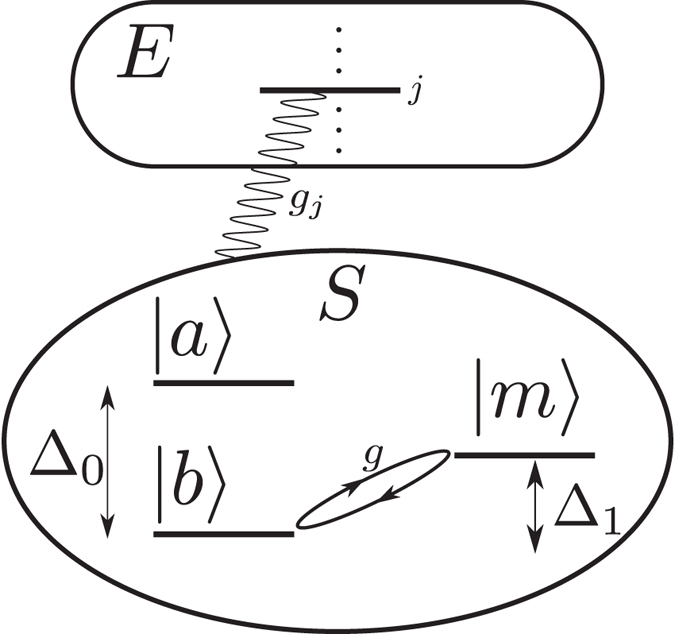
Sketch of our model: A two-level system, with excited and ground states |*a*〉 and |*b*〉, respectively, interacts with a zero-temperature bosonic environment E, while the lower level |*b*〉 is coherently coupled to an external level |*m*〉 with strength *g*. Such a coupling, enables the control of the |*a*〉 → |*b*〉 decay and of memory effects.

Our system is schematically described in Fig. 1. The total Hamiltonian of the system and the environment in the Rotating Wave Approximation (RWA) can be written as


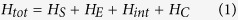


where


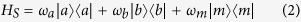



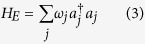



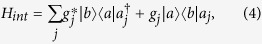



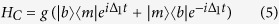


where 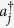 and *a*_*j*_ are the bosonic creation and annihilation operators, *g*_*j*_ and *g* the coupling strength to the *j*th mode of the environment and the level |*m*〉 respectively, *ω*_*a*_, *ω*_*b*_, *ω*_*m*_ the frequencies of the levels |*a*〉, |*b*〉, |*m*〉, *ω*_*j*_ are the frequencies of the environment modes and


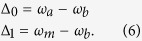


Notice that the level |*m*〉 is neither directly coupled with the environment nor with level |*a*〉; it enters the dynamics for control purposes only. From here on, we will work in the interaction picture defined by the free Hamiltonian. The interaction Hamiltonian *H*_*int*_ + *H*_*C*_ becomes


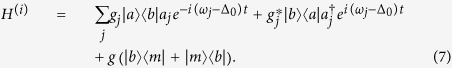


Before going on with our discussion, we briefly comment here on the assumptions required for the RWA to be performed. In fact, the effect of the counter-rotating terms on Quantum Zeno and anti-Zeno effects have been discussed in details in the past few years; see e.g. refs 21,39. It turns out that the RWA description remains approximately valid provided that the coupling with the environment is weak enough and that the state to preserve is the bare excited state of the atomic system, see ref. 21. These will be our assumptions here, that we make in order to discuss in the simplest possible way the interplay between Zeno-type control of the decay and memory effects, which is our main focus. In fact, from the technical point of view, after performing a unitary Frölich-Nakajima transformation, and keeping up to second order terms, the counter-rotating terms can be effectively eliminated and a Hamiltonian identical to that of Eq. (7) is obtained.

For simplicity, thus, we directly employ Eq. (7), and consider the case of having at most one excitation in the whole system initially, by taking the atom to be excited with empty environment modes. This means that the initial state can be written as


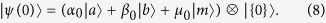


Since the number of excitations is conserved, the state at any later time is


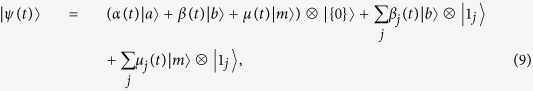


where 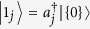 means an excitation in the *j*th mode in the environment. To find the form of the coefficients we solve the Schrödinger equation in the Supplementary Information. For the bosonic environment, we use a Lorentzian spectral density function


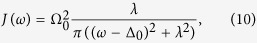


where 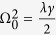. Varying the width of the Lorentzian allows us to use a good and a bad cavity limits – having 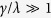 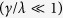 corresponds to good (bad) cavity limit. The solution of the Schrödinger equation is fairly complicated but we can in quite a straightforward manner use it to study the dynamics of the system numerically. In the following section, we first introduce the BLP-measure of non-Markovianity^26^, which is based on the idea of information flow defined in terms of change of trace distance between quantum states. After introducing the measure, we apply it to study the dynamics of our system and present the results.

## Non-Markovianity

In general, quantum channels are one-parameter families {Φ_*t*_)_*t*>0_ of completely positive, trace preserving (CPTP) maps. Each member Φ_*t*_ of the family evolves a quantum state from the initial time *t* = 0 to time *t* > 0, denoted


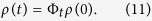


The set of quantum states is the set of positive operators with unit trace and can be endowed with a metric called the trace distance *D*(·, ·) induced by the trace norm ||·||_1_ by the following formula


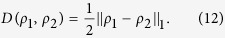


It turns out^40^ that this metric is related to the optimal probability *P*_*max*_ of correctly distinguishing two unknown quantum states from each other. The relation is


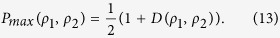


This gives an operational meaning to the trace distance as a measure of distinguishability of quantum states. CPTP maps are contractions for the trace distance^41^, which means that quantum channels tend to decrease the distuinguishability of quantum states. We defined channels as families of CPTP maps that map the input states from initial time *t* = 0 to some later time *t*. If we study the intermediate maps 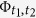, where 0 < *t*_1_ < *t*_2_, which evolve the state from time *t*_1_ to time *t*_2_, the CP property need not hold anymore. This means that locally in time, the trace distance can increase, but never above the original value at time *t* = 0.

Interpreting the decrease of trace distance as information flowing out and increase of the trace distance as a refocusing of information onto the system, we arrive at the Breuer, Laine, Piilo (BLP) measure of non-Markovianity^26^ defined by


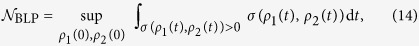


where


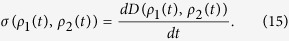


The measure takes as an input a pair of initial states *ρ*_1_(0) and *ρ*_2_(0), monitors the dynamics of their trace distance and adds up all the possible increases in time. This is maximized over all possible choices of the initial pairs giving a value that describes the non-Markovianity of a quantum channel.

The maximization over all possible pairs of initial states seems cumbersome, but can be simplified^42^,^43^. The idea is, that since the state pairs enter the measure only as their difference, the important quantity is actually the direction in the set of quantum states. Many different state pairs give the same direction, meaning that their difference is the same up to a constant. Also, any direction (which here means a traceless hermitian matrix) can be written as a difference of two quantum states^43^. This simplifies the maximization procedure by removing the need to search over all possible state combinations and replacing it with a search over all possible directions, which means that the maximization can be restricted for example to orthogonal state pairs only. We use this method in our numerical work, which is presented in the following section.

## Results

Since the external level |*m*〉 is introduced only to control the dynamics of the original two–level system, we restrict the optimization to initial states where the external level |*m*〉 is empty. This means that we are interested in initial states with *μ*_0_ = 0, which in turn means that the states are described by the parameters *α*_0_ and *β*_0_ alone. This implies that in the initial state density matrix (see the Supplementary Information), only the upper left 2 × 2 block is non-zero, which enables us to represent the interesting ones using the Bloch sphere, i.e. in the form


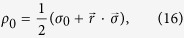


where 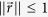 and *σ*_*i*_ are the Pauli matrices. The matrix describing the actual state of our system is the form *ρ*_0_ above, appropriately padded with zeros to make the density matrix 3 × 3. We find the value of the measure by exploiting the direction argument in the following way. First we choose a finite integration time, *λt* = 20, for which the evolution of the system is monitored. A random point and its antipodal point from the Bloch sphere are chosen as the state pair for which the value of the trace distance integral is calculated. This number is stored and the process repeated for a desired number of times (in our case 500 samples). The numbers are normalized to range from 0 to 1. This way we get an approximate map of how the different directions on the Bloch sphere behave in terms of the BLP measure.

**Figure 2:**
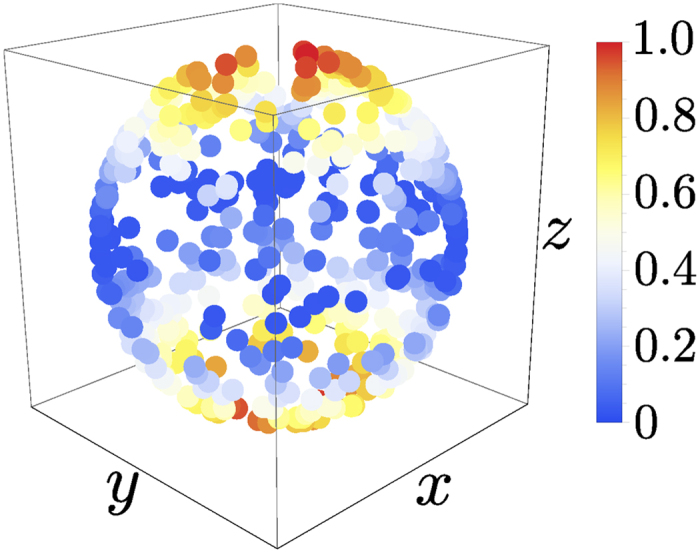
Bloch sphere representation of the directions and the corresponding value for the BLP measure with parameter values *γ* = 10*λ,* g = 10*λ*. Red means high and blue low value for the measure. Note that in the figure, the value of the measure has been normalized so that 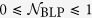.

We study two different regimes – corresponding to a bad and a good cavity – by appropriately choosing the parameter *γ* of the spectral density defined in equation (10). In particular, we take 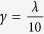 to describe a bad cavity and *γ* = 10*λ* for a good cavity. Also the coupling strength *g* to the external level is varied between 0 and 100*λ*. In all but one of the cases that we studied, the values of the measure were distributed like in Fig. 2, meaning that the maximizing direction is the one through the north and the south pole, corresponding to the initial state pair


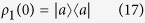



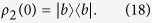


In the one exception, which was the good cavity with zero coupling to the external level, the maximizing pair could have been taken as any pair from the equator.

**Figure 3:**
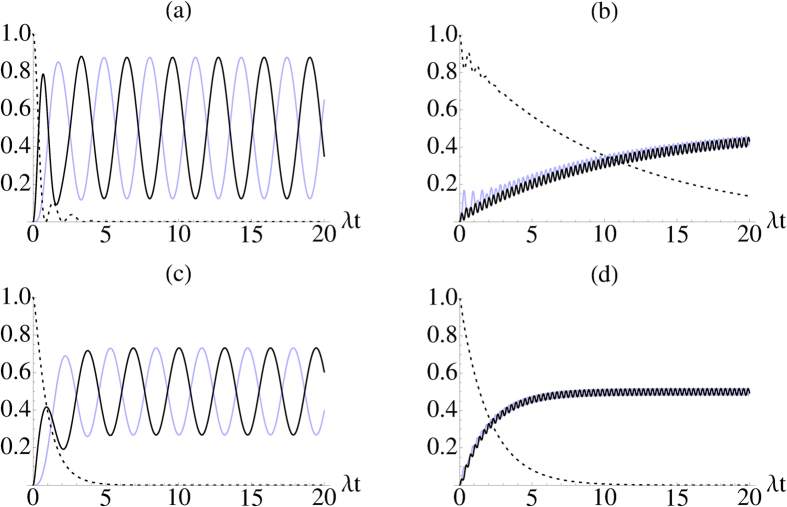
Population of the excited state (black dashed), ground state (black) and the external level (light blue) in the good (**a**,**b**) (*γ* = 10*λ*) and bad (**c**,**d**) cavity 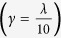 case. In (**a**) we have used *g* = *λ* and in (**b**) *g* = 10*λ*. Similarly in (**c**) 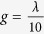 and in (**d**) *g* = *λ*. In all of these cases, we took an initially excited system prepared in |*a*〉.

In Fig. 3, we plot the dynamics of the population of the different levels to show how increasing the coupling *g* affects the dynamics of the system both in good and bad cavity cases. In Fig. 3(a), which corresponds to good cavity with weak coherent coupling between the two lower levels, the excited level population reaches zero followed by a few revival cycles typical for non-Markovian behaviour. The populations of the lower levels keep oscillating with quite a large amplitude and small frequency. When the coupling *g* is increased, Fig. 3(b), the Zeno effect influences the dynamics making the dissipation from the excited state slower. This also decreases the oscillation amplitudes of the populations of the lower levels, and at the same time frequency is higher due to the increased value of *g*. For the bad cavity case in Fig. 3(c) and (d) the situation looks qualitatively similar except that the excited state decreases monotonically in contrast to oscillations displayed by the good cavity case. Due to the monotonic decrease in the excited state population, one would be tempted to conclude that for the bad cavity case the dynamics is Markovian. However, this is not true and eventually it turns out that memory effects influence the dynamics in quite a long time scale even beyond the point when the excited state has already been depleted of population.

**Figure 4:**
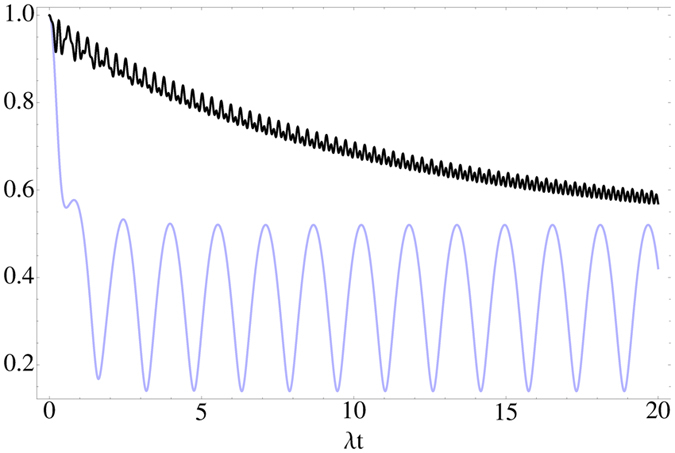
The behavior of the trace distance of the maximizing pair in the good cavity case with *g* = *λ* (light blue) and *g* = 10*λ* (black).

The long term influence of the memory effects is displayed in Fig. 4, which shows the trace distance dynamics for the good cavity case for weak and strong coupling. For the weak coupling, when the Zeno effect does not yet dominate the dynamics, the trace distance keeps oscillating with quite a high amplitude without damping beyond the point when the excited state is already depleted. This looks peculiar since in this regime the system and the environment do not exchange energy anymore. However, this can be explained when looking at the equations of motions and solutions for the various probability amplitudes (for full details, see the Supplementary Information, where an analytic solution of the equations of motion is presented). First, the equation for the excited state amplitude *α* is of the form


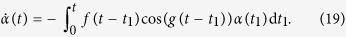


By increasing *g* the kernel of the integral keeps oscillating faster and faster, so that the integral itself decreases, giving rise to a freezing of the excited state amplitude. In general, for an initial state with *β* = *μ* = 0, the open system state at time *t*, is,


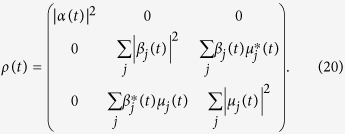


The time evolution of the populations of the two lower levels, also in the regime when excited state is depleted, are given by


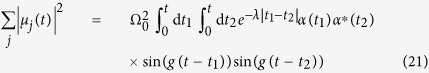



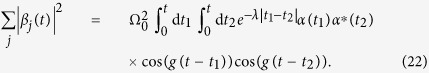


**Figure 5:**
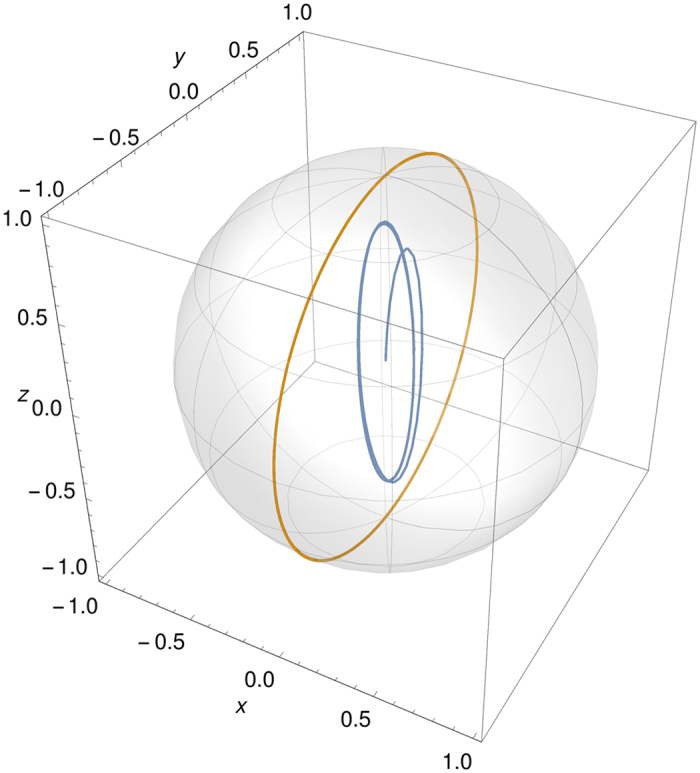
The trajectories of the initial state |*b*〉 (yellow great circle) and |*a*〉 (blue ellipse) in the Bloch sphere representation corresponding to the levels |*b*〉 and |*m*〉 until time *λt* = 20 with parameter values *γ* = 10*λ* and *g* = *λ*. Notice that since all of the population starting from level |*a*〉 is not initially in the system formed by |*b*〉 and |*m*〉, the blue points near the origin (early times) do not correspond to normalized states. The spiral like behavior describes how population enters the system and eventually starts the oscillations that last forever. Note that the plane of rotation is different for the two trajectories which also illustrates why trace distance can oscillate without damping.

The important point to notice here is that even though after some time *α* = 0, the sin and cos terms depend on *t*, and therefore also the values of the integrals depend on time and so do the populations. This is ultimately due to the coupling between the lower levels. However, it makes a large difference, in terms of information flow and non-Markovianity, whether the lower levels are coupled without prior dissipation [see Eqs (30 and 31) in the Supplementary Information], or can enter the coupling cycle after some population has decayed from the excited state. This is depicted in Fig. 5, where we see that the trajectory that the initial state |*a*〉 ends up on is not pure and the plane of rotation around the origin is different than that of the initial state |*b*〉 which remains pure. Moreover, the equations above show how due to the memory effects the lower level populations depend on the past values of the excited state amplitude, and not only on the instantaneous ones, thus explaining the long time survival of oscillations in the trace distance.

The trace distance measure for non-Markovianity is generally associated to a back-flow of information into the open system, so that a question naturally arises: Where does the information come from in this case? To answer, let us consider the total system state as a function of time, for initial states with *β* = *μ* = 0, and after the excited state has decayed, *α* = 0. It is


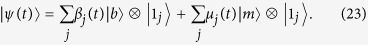


Taking a trace over the system shows that the environmental state does not change anymore. However, the coherences within the total system state do change and also the system-environment correlations. It is this change of the correlations which is ultimately responsible for long-term memory effects here. Note that these memory effects are induced by coherently coupling the second lower to the ground state. In other words, in this case, the origin of the memory effects is not in the engineering of the environment properties, nor in changing the system-environment coupling. Instead, it is related to manipulating the global coherences in the total system, which is enabled by the coherent coupling.

**Figure 6:**
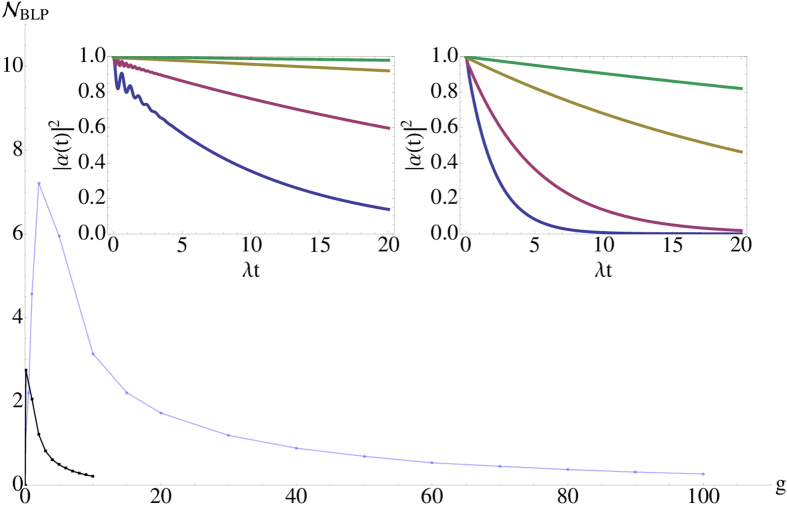
The BLP measure as a function of the coupling strength *g* to the external level. The light blue line is for the good cavity regime (*γ* = 10*λ*), while black refers to the bad cavity case 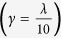. The left inset displays the population of the excited state as a function of time with *g* = 10*λ*, 20*λ*, 50*λ*, 100*λ* (from bottom to top) in the good cavity case. The right inset gives the same in the bad cavity case with *g* = *λ*, 2*λ*, 5*λ*, 10*λ* (from bottom to top).

Let us now turn the attention to the amount of memory effects when increasing the coupling *g* and Zeno effect. The BLP measure for the good and bad cavity cases as a function of *g* is displayed in Fig. 6. The memory effects are more pronounced in the good cavity case than in the bad one. However, in both of the cases the amount of memory effects behaves in non-monotonic way. There is a specific value of *g* where the maximum is reached. This is inherently related to the fact that within the current system, there are two sources of non-Markovianity. Small-time oscillations in the excited state population and long-time persistent oscillations for the ground state populations. When *g* = 0, the bad cavity case does not display memory effects whereas the good cavity case displays minor memory effects. Increasing the coupling constant *g* induces the ground state oscillations with increasing amplitude making the memory effects more prominent. However, at the same time the Zeno-effect tends to freeze excited state population and as a consequence, reduce the ground state oscillations. Therefore, due to the these competing effects, a specific value of *g* allows to maximize the memory effects and beyond this point Zeno-effect begins to dominate reducing non-Markovianity. See also the insets in Fig. 6 displaying in detail how Zeno-effect appears freezing the excited state dynamics.

## Conclusions

We have studied both Zeno-effect and non-Markovianity in a three-level system. The results show that the same coupling, which is used to freeze the excited state dynamics, also induces non-Markovianity in non-trivial manner. In particular, the memory effects persist for long times when the system and the environment do not exchange energy anymore. As a matter of fact, there is a competition between the strength of the memory effects and freezing of the excited state population. As a consequence, the amount of non-Markovianity behaves in non-monotonic way in terms of the strength of Zeno effect. Eventually, when the population dynamics of the excited state completely freezes, memory effects disappear. However, we have revealed a parameter regime which displays a rich interplay between Zeno and non-Markovian dynamics and this also identifies a novel source for memory effects whose origin is inherently independent of the properties of the environment.

## Additional Information

**How to cite this article**: Karlsson, A. *et al*. Quantum Zeno effect and non-Markovianity in a three-level system. *Sci. Rep.* **6**, 39061; doi: 10.1038/srep39061 (2016).

**Publisher's note:** Springer Nature remains neutral with regard to jurisdictional claims in published maps and institutional affiliations.

## Supplementary Material

Supplementary Dataset 1
