# Supplementary material for: Quantum Zeno-type effect and non-Markovianity in a three-level system
Source: Sci Rep. 2016 Dec 20;6:39061. doi: 10.1038/srep39061 (PMC5171700; doi:10.1038/srep39061)
Supplement: Supplementary Dataset 1 [file srep39061-s1.pdf]

SUPPLEMENTARY INFORMATION: QUANTUM ZENO-TYPE EFFECT AND NON-MARKOVIANITY IN A THREE-LEVEL SYSTEM

Let us assume that initially there is only one excitation in the system and that the environment modes are empty. Then the initial state can be written as

$$|\psi(0)\rangle = (\alpha_0 |a\rangle + \beta_0 |b\rangle + \mu_0 |m\rangle) \otimes |\{0\}\rangle . \tag{S.1}$$

Since the excitation number is conserved, the state at any later time is

$$|\psi(t)\rangle = (\alpha(t) |a\rangle + \beta(t) |b\rangle + \mu(t) |m\rangle) \otimes |\{0\}\rangle + \sum_j \beta_j(t) |b\rangle \otimes |1_j\rangle + \sum_j \mu_j(t) |m\rangle \otimes |1_j\rangle , \tag{S.2}$$

where $|1_j\rangle = a_j^\dagger |\{0\}\rangle$ is the state with one excitation in the jth mode of the environment. Equivalently, the state in density matrix form $\rho(t)$ after taking the partial trace over the environmental degrees of freedom from equation (S.2) is

$$\begin{pmatrix} |\alpha(t)|^2 & \alpha(t)\beta^*(t) & \alpha(t)\mu^*(t) \\ \alpha^*(t)\beta(t) & |\beta(t)|^2 + \sum_j |\beta_j(t)|^2 & \beta(t)\mu^*(t) + \sum_j \beta_j(t)\mu_j^*(t) \\ \alpha^*(t)\mu(t) & \beta^*(t)\mu(t) + \sum_j \beta_j^*(t)\mu_j(t) & |\mu(t)|^2 + \sum_j |\mu_j(t)|^2 \end{pmatrix} . \tag{S.3}$$

Schrödinger equation now leads to the following set of coupled differential equations for the coefficients

$$\dot{\beta}(t) = -ig\mu(t) \tag{S.4}$$

$$\dot{\mu}(t) = -ig\beta(t) \tag{S.5}$$

$$\dot{\alpha}(t) = -i \sum_j g_j e^{-i(\omega_j - \Delta_0)} \beta_j(t) \tag{S.6}$$

$$\dot{\beta}_j(t) = -ig_j^* e^{i(\omega_j - \Delta_0)} \alpha(t) - ig\mu_j(t) \tag{S.7}$$

$$\dot{\mu}_j(t) = -ig\beta_j(t). \tag{S.8}$$

From the above equations we can directly solve for two coefficients

$$\beta(t) = \beta_0 \cos(gt) - i\mu_0 \sin(gt) \tag{S.9}$$

$$\mu(t) = \mu_0 \cos(gt) - i\beta_0 \sin(gt). \tag{S.10}$$

To proceed, we use the following transformation to decouple equations (S.7) and (S.8)

$$l_j(t) = \frac{\beta_j(t) + \mu_j(t)}{\sqrt{2}} \qquad r_j(t) = \frac{\beta_j(t) - \mu_j(t)}{\sqrt{2}}, \tag{S.11}$$

which leads to differential equations that can be integrated directly

$$\dot{l}_j(t) = -i\frac{g_j^*}{\sqrt{2}} e^{i(\omega_j - \Delta_0)t} \alpha(t) - igl_j(t) \tag{S.12}$$

$$\dot{r}_j(t) = -i\frac{g_j^*}{\sqrt{2}} e^{i(\omega_j - \Delta_0)t} \alpha(t) + igr_j(t). \tag{S.13}$$

Integrating the above equations and solving for β_j and μ_j from equation (S.11) yields

$$\beta_j(t) = -ig_j^* \int_0^t \mathrm{d}t_1 e^{i(\omega_j - \Delta_0)t_1} \cos(g(t - t_1))\alpha(t_1) \tag{S.14}$$

$$\mu_j(t) = -ig_j^* \int_0^t \mathrm{d}t_1 e^{i(\omega_j - \Delta_0)t_1} \sin(g(t - t_1))\alpha(t_1). \tag{S.15}$$

Now we can insert the solution for β_j to the differential equation for α, which leads to

$$\dot{\alpha}(t) = -\int_0^t \sum_j |g_j|^2 e^{-i(\omega_j - \Delta_0)(t-t_1)} \cos(g(t-t_1))\alpha(t_1)\mathrm{d}t_1. \tag{S.16}$$

To proceed from here we approximate the state of the environment with a continuous distribution of modes, whose spectral density is given by the function $J(\omega)$. The approximation amounts to the replacement

$$\sum_j |g_j|^2 e^{-i(\omega_j - \Delta_0)(t-t_1)} \to \int_{-\infty}^{\infty} J(\omega) e^{-i(\omega-\Delta_0)(t-t_1)}\mathrm{d}\omega \equiv f(t-t_1). \tag{S.17}$$

With this, the equation for α becomes

$$\dot{\alpha}(t) = -\int_0^t f(t-t_1)\cos(g(t-t_1))\alpha(t_1)\mathrm{d}t_1. \tag{S.18}$$

Let us assume that the form of the spectral density is a Lorentzian

$$J(\omega) = \Omega_0^2 \frac{\lambda}{\pi((\omega-\Delta_0)^2+\lambda^2)}, \tag{S.19}$$

where $\Omega_0^2 = \frac{\lambda\gamma}{2}$. The form of the spectral density function could be almost anything, but this choice makes the calculations fairly simple. By controlling the parameters γ and λ, which are basically the height and width of the Lorentzian, we can switch between Markovian and non-Markovian behavior of the system. We can now evaluate the integral in equation (S.17) and obtain

$$f(t-t_1) = \Omega_0^2 e^{-\lambda|t-t_1|}. \tag{S.20}$$

Let us denote

$$F(t-t_1) = \Omega_0^2 e^{-\lambda|t-t_1|}\cos(g(t-t_1)) \tag{S.21}$$

and denote $t - t_1 = \tau$. Then the Laplace transform of F is

$$\tilde{F}(s) = \int_0^\infty e^{-s\tau}F(\tau)\mathrm{d}\tau = \Omega_0^2 \frac{s+\lambda}{(s+\lambda)^2+g^2}. \tag{S.22}$$

Laplace transforming the differential equation for α we get

$$s\tilde{\alpha}(s) - \alpha_0 = -\tilde{F}(s)\tilde{\alpha}(s), \tag{S.23}$$

which combined with equation (S.22) leads to

$$\tilde{\alpha}(s) = \alpha_0 \frac{(s+\lambda)^2+g^2}{s(s+\lambda)^2 + s(\Omega_0^2+g^2) + \Omega_0^2\lambda}. \tag{S.24}$$

Denoting the three roots of the denominator with s_i and taking the inverse transform, we solve for

$$\alpha(t) = \alpha_0\Big(\frac{(s_1+\lambda)^2+g^2}{(s_1-s_2)(s_1-s_3)}e^{s_1 t} + \frac{(s_2+\lambda)^2+g^2}{(s_2-s_1)(s_2-s_3)}e^{s_2 t} + \frac{(s_3+\lambda)^2+g^2}{(s_3-s_1)(s_3-s_2)}e^{s_3 t}\Big). \tag{S.25}$$

With this solution and after some simplifications we finally solve for the remaining coefficients in the density matrix

$$\sum_j |\mu_j(t)|^2 = \Omega_0^2 \int_0^t \mathrm{d}t_1 \int_0^t \mathrm{d}t_2 e^{-\lambda|t_1-t_2|}\alpha(t_1)\alpha^*(t_2)\sin(g(t-t_1))\sin(g(t-t_2)) \tag{S.26}$$

$$\sum_j |\beta_j(t)|^2 = \Omega_0^2 \int_0^t \mathrm{d}t_1 \int_0^t \mathrm{d}t_2 e^{-\lambda|t_1-t_2|}\alpha(t_1)\alpha^*(t_2)\cos(g(t-t_1))\cos(g(t-t_2)) \tag{S.27}$$

$$\sum_j \mu_j^*(t)\beta_j(t) = \Omega_0^2 \int_0^t \mathrm{d}t_1 \int_0^t \mathrm{d}t_2 e^{-\lambda|t_1-t_2|}\alpha(t_1)\alpha^*(t_2)\cos(g(t-t_1))\sin(g(t-t_2)). \tag{S.28}$$
